# Parental health risk perceptions and preventive measures related to Children’s second-hand cigarette smoke exposure in Malaysia

**DOI:** 10.1186/s12889-021-11825-2

**Published:** 2021-10-15

**Authors:** Suria JUNUS, Chii-Chii CHEW, Pravin SUGUNAN, Nurul-Fazlin MEOR-AZIZ, Nurul Ain ZAINAL, Husna Mat HASSAN, Mazetty-Aiza ABU-MANSOR, Hazirah ABU-ZAMRI, Amar-Singh HSS

**Affiliations:** 1 Clinical Research Centre, Hospital Raja Permaisuri Bainun, Ministry of Health, Jalan Raja Ashman Shah, 30450 Ipoh, Perak Malaysia; 2 Paediatric Department, Hospital Raja Permaisuri Bainun, Ministry of Health, Ipoh, Perak Malaysia; 3 Institute of Training Ministry of Health, Ministry of Health, Ulu Kinta, Perak Malaysia

**Keywords:** Secondhand smoking, Child, Parents, Awareness, Perception, Preventive measures

## Abstract

**Background:**

Secondhand smoke (SHS) exposure can affect physical development in children. An understanding of parental risk perception of SHS could guide efforts to develop measures for prevention of SHS exposure among children. This study aimed to assess parental risk perceptions of SHS and action taken by parents to minimise SHS exposure in their children.

**Methods:**

This cross-sectional nationwide study conducted in 2018 recruited convenience sample of 289 parents with children up to age 12 at public areas. Parents were asked to rate the risk level from 1 (no risk) to 5 (extremely high risk) by looking at photographs of an adult smoking in the presence of a child in 8 different situations. The implementation of smoking restriction rules was assessed. Mean scores were calculated with higher scores representing higher risk perception of SHS to child’s health. Linear regression analysis was used to determine factors associated with the level of parental risk perception of SHS exposure to their children’s health.

**Results:**

A total of 246 parents responded. Their mean age was 35 years (SD 6.4). The majority were mothers (75.6%), Malays (72.0%) and had tertiary education level (82.5%), and non-smoker (87.1%). The mean age of respondents’ youngest child was 3 years (SD 3.1). The risk perception level was high [mean scores: 4.11 (SD: 0.82)]. Most parents implemented household (65.0%) and car (68.3%) smoking restriction rules. Lower levels of risk perception were observed among participants who were current smokers (*p* < 0.001), lived with a smoker (*p* < 0.001), allowed household smoking with an open window (*p* = 0.027).

**Conclusion:**

Most parents perceived that risks of SHS exposure to their children were high but only two-thirds of them had set rules prohibiting smoking. Health policymakers should pay attention to factors associated with lower risk perception among parents.

**Trial registration:**

This study was approved by the Medical Research Ethics Committee, Ministry of Health Malaysia (Registration Number: NMRR-18-3299-44967).

## What is already known on this topic?

1. There is no risk-free level for exposure of children to secondhand smoke.
2. Secondhand smoke harms children and adults, and the only way to fully protect non-smokers is to eliminate smoking in homes, worksites, and public places.
3. Separating smokers from non-smokers, opening windows, or using air filters does not prevent people from breathing secondhand smoke.

## What does this study add?

1. The majority of parents were non-smokers and perceived that the risk of secondhand smoke (SHS) exposure to their children was high. Most parents perceived that cigarette smoke exposure in an enclosed space such as a kitchen had the highest health risk to the children.
2. Worryingly, almost one-third of the participants did not set smoking restriction rules inside their house or car. Nevertheless, most of them had taught their children to stay away from smokers.
3. Parents who were current smokers, allowed smoking in the house with an open window, and the presence of another household member who was a smoker were factors significantly associated with a lower risk perception of secondhand smoke exposure to children. Policymakers should consider focusing efforts to raise awareness on parents with these characteristics to achieve maximal effect.

## Introduction

Second-hand smoke exposure (SHS) is defined as a person’s involuntary exposure to tobacco smoke generated by a smoker or by the burning of tobacco products. SHS has deleterious effects on health, as the smoke contains 70 types of carcinogens [1], and 2,500,000 non-smokers have died or suffered adverse health-related outcomes following SHS exposure between 1964 and 2014 [2, 3]. The economic burden of the morbidity and mortality from second-hand smoke in children and adults is amounts to USD $267 million annually [4]. The harmful effects observed in children include an increased risk of respiratory symptoms and infections and a reduced rate of lung growth [5]. Children are particularly vulnerable to the harmful effects of SHS because of their growing bodies and faster breathing rates compared to adults. Approximately 40% of children worldwide are exposed to SHS [6].

Parental health risk perceptions influence their health-related behaviour and potential interventions to influence behaviour. Parents who perceive higher health risks of SHS exposure are more likely to prohibit smoking in their homes or cars [7]. Parental risk perception is important for developing effective interventions to protect children from SHS [8]. Increasing parental risk awareness by educating parents about the dangers of SHS could sensitise them to the harmful health effects attributed to SHS [9, 10].

Socio-demographic characteristics, including ethnicity, children’s age and parental smoking behaviour, are associated with parental risk perception of SHS exposure in children [9, 11]. Parents who perceive a higher risk of SHS exposure tend to be more protective and avoid smoking at home. In Malaysia, adolescents of Malay ethnicity and descendants of natives of Sabah and Sarawak are more likely to experience SHS exposure compared to those of Chinese and Indian ethnicity [12]. Smokers with children younger than 3 years tend to be stricter in avoiding SHS exposure compared to those with older children [10]. SHS exposure is 3.5 times more likely when there is a smoker in the family [13]. It is essential to determine the factors that influence the risk perception of SHS exposure amongst parents to develop effective interventions for specific groups of parents with low risk perception, which may be more effective than blanket interventions targeting all parents [9, 10].

Smoking bans and legislation have proven most effective in reducing SHS exposure [5] However, these rules are impossible to enforce in the home and inside private vehicles. In the United States, household smoking restrictions increased from 69.3 to 79.5% and car smoking prohibitions increased from 68.3 to 81.8% [14]. Less than half (40.9%) of the Malaysian population have adopted household smoking restriction rules [15], and parental perspectives on this practice have yet to be explored in Malaysia. A lower rate of household smoking restriction was reported by Malaysian adolescents who lived with smoking parents (67%); less than two-thirds (60.8%) of the households applied this rule at home [16]. Malaysian adolescents reported that the prevalence of SHS exposure inside the car of their parents/guardians in the past week was 23.3% [12]. The extent of smoking prohibitions in cars among Malaysian parents has not been established.

Unfortunately, there is evidence that interventions aimed at parental smoking cessation are ineffective in reducing SHS exposure among children [17]. It is vital for parents to enforce rules banning anyone, including themselves, from smoking in the vicinity of their children [18]. Gauging the perspectives of parents of young children in relation to such rules could help ascertain the feasibility of these interventions. In view of the great variations in socio-demographic characteristics, ethnic groups and smoking behaviour amongst parents in Malaysia, this study aimed to determine the health risk perceptions of parents with regard to SHS exposure amongst their children and their current practices aimed at protecting children from SHS exposure.

## Methodology

### Study design & setting

This cross-sectional nationwide study conducted from October 2018 until April 2019 involved data collection from 13 states and 2 federal territories in Malaysia. Eligible participants were recruited in public areas, including shopping malls, playgrounds and food courts. The data collectors approached parents at their convenience in the abovementioned public areas during weekends. The exact time of data collection was not specified.

### Population & sample size

The study included parents with at least one child aged ≤12 years. The sample size was calculated based on an estimated smoking prevalence amongst Malaysian adults of 25% (highest estimate reported by the National Health and Morbidity Survey Report in 2015) [19]. A minimum sample size of 289 parents was required, with a precision level set at 5% and a confidence level of 95%. Twenty participants were needed from each state and federal territory.

### Instrument

A validated questionnaire measuring parental risk perception of SHS on their children’s health was adapted from Myers et al. [8]. The instrument had a good reliability coefficient, with a Cronbach’s alpha of 0.934 and a validated construct to quantify parents’ perceptions of their children’s exposure to SHS. Due to logistical difficulties in collecting nationwide data, the third section of the questionnaire with eight photographs was adapted. Following consultation with the author, it was felt that this adaptation did not violate the questionnaire’s validity. The photographs showed parents smoking in the presence of young children in various settings (indoors with an open window, in a closed room and outdoors) at different distances. The exact question asked was ‘Please rate the health risk to the child in the pictured situation’. The respondents were required to rate the risk level of SHS exposure to young children’s health based on a 5-point Likert scale ranging from 1 (no risk) to 5 (extremely high-risk). A questionnaire from Ratajczak et al. (2018) was adapted to assess if parents had implemented household and car smoking restriction rules as preventive measures to protect their children from SHS exposure [20]. An additional question was added as a separate section to explore whether parents had taught their children to stay away from smokers as a measure to avoid SHS exposure [18].

The questionnaire was translated into Malay via forward and backward translation. The questionnaires in English and Malay underwent content and face validation by 2 paediatricians and were pre-tested by 10 parents for each language. Both the English and Malay questionnaires were built into an online electronic form (Google Forms).

### Data collection

Data collectors from each state were sent instructions specifying the procedures. If both parents were present during recruitment, they were invited to participate and required to answer the questionnaire separately, which allowed each parent to respond without being influenced by the other. Both parents from the same household were allowed to participate mainly because of the issue of gender imbalance in the context of SHS exposure at home. Women may sometimes demonstrate behaviour that meets cultural social expectations, particularly in Asian countries. Some women think that it is inappropriate to interrupt the husband or father-in-law who smokes in the shared space at home despite having a negative attitude towards SHS. A qualitative review found a gender imbalance, describing women’s lack of agency in changing male family members’ smoking behaviour in the home [21, 22]. Hence, the mother’s perception may be different from the father’s even though they are from the same household. The parents who agreed to participate were required to give online informed consent and answer the questionnaire on a mobile device provided by the data collector to ensure only one entry per participant.

### Data analysis and interpretation

All statistical tests were analysed using SPSS version 20 (IBM Corp. Released 2011. IBM SPSS Statistics for Windows, Version 20.0. Armonk, NY: IBM Corp). Parental occupation was coded based on Standard Occupational Classification 2010 [23]. Descriptive analysis was used to summarise participants’ characteristics. Each scenario of SHS exposure was treated as equal risk based on the statement of ‘there is no risk-free level of secondhand smoke exposure; even brief exposure can be harmful to health’ [1]. An overall mean SHS risk perception index was calculated for the 8 individual SHS exposure measures. The overall mean risk perception index ranged from 1 (no risk) to 5 (extremely high risk) with higher scores representing higher parental perceived risk [8]. Simple linear regression analysis was used to identify measures potentially associated with overall mean risk perceptions (*p*-value < 0.05). A multivariable linear model was then estimated using backward elimination to identify statistically significant variables for a final multivariable model. Variables included in the final multivariable regression model that predicted SHS risk perception index were parental role, education level, mother’s occupation, father’s occupation, presence of an extended family member in the household who was a smoker, whether children were taught to stay away from smokers, presence of household and car rules allowing or prohibiting smoking. Assumptions were checked, no multicollinearity problems were detected and a *p*-value of less than 0.05 was considered statistically significant.

## Results

A total of 246 parents participated, with a response rate of 85.1%. The mean age of the respondents was 35 years (SD 6.4); most were mothers (75.6%), of Malay ethnicity (72.0%) and had completed tertiary education (82.5%). More than half of the participants had intermediate-level occupations [mothers (64.2%) and fathers (52.8%)], which were defined as jobs that do not involve general planning or supervisory roles [23]. Most of the parents had more than one child at home (76.0%), with a maximum of 12 children reported by one respondent. The mean age of the youngest child was 3 years (SD 3.1) (Table 1).

**Table 1:** Characteristics of the participants

Variables		*n* = 246, n (%)
Parent’s age, *mean (SD)*		35 (6.4)
Parental role	Mother	186 (75.6)
	Father	60 (24.4)
Ethnicity	Malay	177 (72.0)
	Bumiputera Sabah	22 (8.9)
	Chinese	20 (8.1)
	Indian	16 (6.5)
	Bumiputera Sarawak	7 (2.8)
	Others	4 (1.6)
State residing	Selangor	39 (15.9)
	Perak	34 (13.8)
	Johor	25 (10.2)
	Melaka	24 (9.8)
	Kelantan	22 (8.9)
	Penang	11 (4.5)
	Pahang	8 (3.3)
	Perlis	8 (3.3)
	Negeri Sembilan	6 (2.4)
	Kedah	3 (1.2)
	Terengganu	4 (1.6)
	Sabah	24 (9.8)
	Sarawak	19 (7.7)
	Federal Territory of Kuala Lumpur	10 (4.1)
	Federal Territory of Labuan	9 (3.7)
Education Level	Secondary	43 (17.5)
	Tertiary	203 (82.5)
Mother’s Occupation^a^	Managerial and professional	9 (3.7)
	Intermediate*	158 (64.2)
	Small employer/ own company	7 (2.8)
	technical	9 (3.7)
	Semi-routine/ routine	14 (5.7)
	Never worked/ unemployed	49 (19.9)
Father’s Occupation^a^	Managerial and professional	9 (3.7)
	Intermediate*	130 (52.8)
	Small employer/ own company	25 (10.2)
	technical	40 (16.3)
	Semi-routine/ routine	32 (13.0)
	Never worked/ fulltime unemployed	10 (4.1)
Parent’s smoking status	Current smoker	17 (6.8)
	Former smoker	15 (6.1)
	Never smoked	217 (87.1)
Mothers’ smoking status	Current	0 (0.0)
	Former	1 (0.5%)
	Never	185 (99.5)
Fathers’ smoking status	Current	17 (28.3)
	Former	14 (23.3)
	Never	29 (48.3)
Living with extended family member who is an active smoker		83 (33.7)
Number of children at home	1	59 (24.0)
	2	89 (36.2)
	3	46 (18.7)
	4	29 (11.8)
	5	17 (6.9)
	6	4 (1.6)
	9	1 (0.4)
	12	1 (0.4)

^a^ The National Statistics Socio-economic Classification rebased on Standard Occupational Classification 2010 (SOC 2010)

*Intermediate occupations include clerical, sales, services, and technical occupations that do not involve general planning or supervisory powers (SOC 2010)

### Non-smoker parents

Most respondents (87.1%) were non-smokers. Approximately one-third (33.7%) had a tobacco cigarette smoker living in the same household (Table 1).

### Parents’ smoking status

None of the mothers recruited were current smokers and only one mother (0.5%) identified as a former smoker. Among fathers, 17 (28.3%) currently smoked and 14 (23.3%) were former smokers, comparatively higher than the mothers group (Table 1).

### Parental health risk perception

Overall, the mean score for parental risk perception was 4.11 (SD: 0.82), indicating a perception of SHS exposure as a high risk to their children’s health. A varying proportion of participants (ranging from 35.4 to 59.3%) rated most of the situations given as posing an extremely high risk of SHS exposure to the child’s health. A rating of ‘extremely high-risk’ was given most frequently by participants for a situation involving an adult smoking in the presence of a child in the kitchen (59.3%) (Photograph 3), followed by an adult smoking inside a car with the window open while a child was sitting in the rear (54.1%) (Photograph 5).

Roughly equal numbers of participants gave ‘high-risk’ (33.7%) and ‘extremely high-risk’ (35.4%) ratings for a situation demonstrating an adult smoking on a balcony with an open door while children were playing inside the house (Photograph 7). Two situations which most parents perceived as a ‘high risk’ to their children’s health involved an adult smoking in the presence of a child at a playground (42.3%) (Photograph 2) and an adult smoking some distance away from a child outdoors (34.1%) (Photograph 4). A rating of ‘no risk’ was given most frequently by the participants for Photograph 4 (8.1%) compared to the other situations (ranging from 0.8 to 2.8%) (Table 2).

**Table 2:** Parental risk perception of SHS exposure to their children’s health

Variables	*n* = 246, n (%)					Overall mean SHS risk perception score (SD)
	No risk	Low risk	Moderate risk	High risk	Extremely high risk	
Photograph 1	5 (2.0)	12 (4.9)	13 (5.3)	98 (39.8)	118 (48.0)	4.27 (0.92)
Photograph 2	3 (1.2)	17 (6.9)	24 (9.8)	104 (42.3)	98 (39.8)	4.13 (0.93)
Photograph 3	2 (0.8)	3 (1.2)	15 (6.1)	80 (32.5)	146 (59.3)	4.48 (0.74)
Photograph 4	20 (8.1)	24 (9.8)	67 (27.2)	84 (34.1)	51 (20.7)	3.50 (1.16)
Photograph 5	2 (0.8)	16 (6.5)	16 (6.5)	79 (32.1)	133 (54.1)	4.32 (0.92)
Photograph 6	6 (2.4)	16 (6.5)	23 (9.3)	83 (33.7)	118 (48.0)	4.18 (1.01)
Photograph 7	7 (2.8)	23 (9.3)	46 (18.7)	83 (33.7)	87 (35.4)	3.89 (1.08)
Photograph 8	5 (2.0)	16 (6.5)	26 (10.6)	94 (38.2)	105 (42.7)	4.13 (0.98)

### Actions to protect children from SHS

About two-thirds of the parents (65.0%) had implemented rules prohibiting smoking inside their houses, and only 3.3% of the parents allowed smokers to smoke indoors with a window open. About 68.3% of parents forbade smoking inside the car when travelling with children. However, 6.9% of parents allowed smoking inside the car with a window open. Despite many parents setting rules prohibiting smoking in their homes or cars, there was no statistically significant association between the parental mean scores of SHS risk perception and their behaviour in setting smoking prohibition rules. The gender of the parents did not moderate the relationship between perceived risk and having smoking ban rules (Table 3). Most respondents (85.4%) had taught their children to stay away from cigarette smokers.

**Table 3:** Mean scores of risk perception associated with home/car smoking ban

Variables		*OR*	(95% CI *OR*)	*χ*^2^ stat. (df)	*p*-value*
Mean score of risk perception	Home Smoking Ban	1.31	0.95, 1.81	2.757 (1)	0.097
	Car Smoking Ban	1.08	0.77, 1.53	0.202 (1)	0.653
Mothers’ mean score of risk perception	Home Smoking Ban	1.42	0.86, 2.34	1.867 (1)	0.172
	Car Smoking Ban	1.31	0.77, 2.24	0.982 (1)	0.322
Fathers’ mean score of risk perception	Home Smoking Ban	1.28	0.79, 2.09	1.006 (1)	0.316
	Car Smoking Ban	0.88	0.52, 1.49	0.247 (1)	0.619
Interaction of Parents’ gender with risk perception	Home Smoking Ban	1.00	0.85, 1.18	0.002 (1)	0.963
	Car Smoking Ban	0.95	0.80, 1.13	0.279 (1)	0.598

*analysed with single binary logistic regression. *OR* Odd Ratio, *CI* Confidence interval

### Factors associated with parental health risk perception of SHS exposure

The mothers who worked in professional (β = 0.60, *p* = 0.012), intermediate (β *=* 0.37, *p* = 0.001), and semi-routine occupations (β *=* 0.61, *p* = 0.014), and participants who had taught their children to stay away from smokers (β *=* 0.42, *p* = 0.001) and set rule that ban smoking inside the house (β *=* 0.19, *p* = 0.040), had significantly higher scores in risk perception than the comparison groups. The participants who being a current smoker (β *=* − 0.93, *p* < 0.001), lived with smokers (β = − 0.33, *p* < 0.001) and those who allowed household smoking with an open window (β *= −* 0.55, *p* = 0.027) were associated with lower risk perception scores (Table 4).

**Table 4:** Factors associated with parental risk perception of SHS exposure to their child’s health

	Simple Linear Regression				Multiple Linear Regression			
Variables	Crude β	95% CI	t-statistics	*p*-value	Adj. β^§^	95% CI	t-statistics	*p*-value
Parent’s age	0.01	−0.01,0.02	0.551	0.582	–	–	–	–
Parental role								
Mother	–	–	–	–				
Father	−0.72	− 0.94,-0.49	−6.334	**< 0.001**	− 0.25	− 0.50, 0.01	−1.826	0.057
Ethnicity								
Others^ƚ^	–	–	–	–	–	–	–	–
Malay	0.18	−0.05, 0.41	1.523	0.129				
Education Level								
Secondary	–	–	–	–	–	–	–	–
Tertiary	0.48	−0.21, 0.74	3.526	**0.001**				
Mother’s Occupation^a^								
Never worked	–	–	–	–				
Managerial & professional	0.40	−0.15, 0.95	1.445	0.150	0.60	0.13, 1.06	2.537	**0.012**
Intermediate	0.32	−0.10, 0.53	2.929	**0.004**	0.37	0.15, 0.59	3.287	**0.001**
Small employer/own company	−0.21	− 0.83, 0.41	−0.660	0.510	0.27	−0.24, 0.79	1.040	0.300
Lower supervisory/technical	0.24	−0.31, 0.79	0.872	0.384	0.37	−0.10, 0.83	1.540	0.125
Semi-routine/ routine	0.33	−0.12, 0.77	1.444	0.150	0.61	0.21, 1.00	2.488	**0.014**
Father’s Occupation^a^								
Never worked	–	–	–	–	–	–	–	–
Managerial & professional	0.37	−0.18, 0.92	1.341	0.181				
Intermediate	0.19	−0.02, 0.40	1.811	0.071				
Small employer/own company	−0.84	−1.16,-0.51	−5.077	**< 0.001**				
Lower supervisory/technical	−0.14	− 0.41,0.15	− 0.950	0.343				
Semi-routine/ routine	0.23	−0.07, 0.54	1.508	0.133				
Parent’s smoking status								
Never smoked	–	–	–	–	–	–	–	**–**
Current smoker	−1.64	− 1.99,-1.29	−9.191	**< 0.001**	− 0.93	− 1.35, 0.51	−4.395	**< 0.001**
Former smoker	−0.24	− 0.68, 0.19	−1.118	0.265	− 0.04	− 0.43, 0.36	− 0.181	0.857
Number of children	−0.03	− 0.10, 0.04	− 0.757	0.450	–	–	–	–
Lived with an extended family member who was a smoker	−0.43	− 0.64,-0.22	− 3.973	**< 0.001**	− 0.33	− 0.51, − 0.15	−3.608	**< 0.001**
Have taught child to stay away from smokers	0.76	0.49,1.04	5.443	**< 0.001**	0.42	0.17, 0.66	3.386	**0.001**
Set household rules for smoking								
Never set rules	–	–	–	**–**	–	–	–	**–**
Allowed household smoking with open window	−.1.13	−1.70,-0.57	−3.957	**< 0.001**	−0.55	− 1.03, − 0.06	−2.224	**0.027**
Prohibited smoking inside the house	0.34	0.12, 0.55	3.121	**0.002**	0.19	0.01, 0.37	2.070	**0.040**
Set smoking rules inside the car								
Never set rules	–	–	–	**–**	–	–	–	**–**
Allowed smoking inside the car with open window	−1.39	− 1.76,-1.03	− 7.467	**< 0.001**				
Prohibited smoking inside the car	0.46	0.25,0.68	4.237	**< 0.001**				

^§^ Adjusted regression coefficient analyzed by Multiple linear regression, F (12, 232) = 14.217, *p* < 0.001, *R*^*2*^ = 0.423. Intercept: 3.667

*CI* confidence interval, Bold *p-*value: statistically significant.

^ƚ^ (Bumiputera Sabah, Chinese, Indian, Bumiputera Sarawak)

^a^ The National Statistics Socio-economic Classification rebased on Standard Occupational Classification 2010 (SOC2010)

## Discussion

Although the parents were aware of the high risk of SHS exposure to their children’s health, the implementation of household and car smoking restriction rules remained inadequate. Interestingly, the parents were more likely to advise their children to stay away from SHS than to set rules against smoking in their homes or cars.

The respondents who were smokers and lived with an extended family member who was an active smoker had lower risk perceptions of SHS exposure to their children’s health, consistent with the findings of other studies conducted in Malaysia and Indonesia. Children living with a father who smoked and who had an extended family member in the same household who was a smoker were more likely to suffer SHS exposure [24, 25]. A Malaysian study in 2011 found that children with these family characteristics had higher levels of GM cotinine concentration (0.71 ng/mL) compared to children living in a non-smoking family (0.32 ng/mL) [24]. The low parental risk perception found in this study suggests that the problem of SHS exposure in children may not have improved in the intervening years and that efforts to increase parental awareness remain inadequate. Our study suggests that specific attention should be paid to increasing awareness amongst parents about active smokers in their households.

Lower parental risk perception was also seen amongst parents who allowed smoking in their homes with an open window, consistent with the findings of Myers et al. [11]. Up to 52% of smoker parents report a lack of belief or awareness about SHS-related adverse health effects in children [26]. Ignorance of the fact that there is no risk-free level of SHS exposure in children could be the reason some parents continue to allow smoking inside the house, as seen in this study [1]. It has been demonstrated that despite preventive measures being taken by parents living with a smoker to protect their children from SHS exposure, the biomarker of SHS exposure detected in children was 5 to 7 times higher than children living in non-smoking households [24, 27]. Current evidence indicates that only a strict total household smoking ban can effectively protect children from SHS [5, 28]. Awareness of this fact must be increased, especially amongst parents living with smokers in the same household.

Household smoking restriction rules have been proposed as an effective way to minimise SHS exposure amongst children [27]. Smoking restriction rules were practised by approximately two-thirds of the respondents, similar to that reported in a 2014 study involving Malaysian adolescents, in which 60.8% of households implemented such rules [16]. The fact that this rate has remained unchanged over the years should warrant increased attention from public health policymakers to enhance awareness among parents on this important matter.

The parents have overall high risk perception of SHS exposure but smoking ban rules at home and car remain inadequately implemented. This findings could be explained by lower risk perception of SHS exposure amongst the fathers as compared to that of the mothers. Gender imbalance in the context of SHS exposure in Asian culture may cause women to find it inappropriate to interrupt the husband or father-in-law who smokes in the shared space at home, even if these women have a negative attitude towards SHS [21, 22]. Therefore, the mothers may consider that it is not likely to set smoking ban rules in the home or car even though they perceive risk of SHS exposure in their children is high.

### Limitations

Despite this study having covered every state in Malaysia, the population recruited might not adequately represent the proportion of all ethnic groups in this country, as most of the respondents were Malay (72%). Malaysia comprises a population of approximately 69% Bumiputera (Malays and indigenous people including *Orang Asli*, Sabah and Sarawak Bumiputera), 23% Chinese, 6.9% Indians and 1.0% other ethnic groups, as at 2018 [29]. The actual risk perception among parents could be lower, as less than 7% of the parents recruited were smokers, which is lower than the prevalence (25%) reported at the national level [19]. Hence, the findings may not accurately represent the risk perceptions of all parents in the Malaysian population. For example, relatively few women in Malaysia smoke compared to men (1.4% vs 43%, respectively) [19]. In the current study, no mothers reported smoking and only one reported being a former smoker. Therefore, the over-representation of mothers in this study might explain the lower prevalence of smokers in this study. It is possible that the prevalence of smokers was associated with parental status in this study. Thus, future studies may explore the risk perception of child exposure to SHS among Malaysian adults who do not have children.

This study did not specify the number of respondents were married; it was not possible to identify individuals from the same household if the couple were recruited by different data collectors. Also, assuming observations were independent may result in *p*-values that are too small, which could influence the conclusions. However, we estimated that few respondents in this study were married couples; data were obtained individually, and each respondent was analysed independently.

### Implications for health policymakers

Factors associated with lower parental risk perception of SHS exposure to child health are crucial for public health policymakers to consider when designing strategies to protect children from SHS. It is important for parents to be aware of the risk of SHS exposure amongst children to avoid adverse effects on their children’s growth and wellbeing. Public health authorities should raise awareness amongst parents by educating them about the harms of SHS exposure to their children and consider implementing rules prohibiting smoking inside houses and cars in the presence of children. For maximal effect, awareness and advocacy measures should specifically target parents who were active smokers and parents who live with an active smoker in the same household.

## Conclusion

The study findings amongst the families surveyed show a general lack of clear rules prohibiting smokers from smoking in the presence of children, even in enclosed areas or inside a car. Not all households, even those who perceived the risk of SHS to be high, had rules against smoking in their homes or cars. Some parents allowed smokers to smoke with open windows in the house or when travelling with children in the car, practices that could expose children to SHS. Parents who were active smokers, the presence of an active smoker in the family and lack of rules prohibiting household smoking were significantly associated with a lower parental risk perception of SHS exposure towards their children’s health. Health policymakers could consider targeting parents with these characteristics when designing programmes to increase awareness of SHS exposure risks amongst children. Such programmes could educate parents regarding the risk of SHS exposure amongst children in various situations and emphasise the importance of implementing strict smoking restriction rules.

## Data Availability

The datasets generated during the current study are not publicly available due to the need for confidentiality but are available from the corresponding author on reasonable request.

## Abbreviations

SHS: Secondhand smoke
GM cotinine: Geometric mean salivary cotinine concentrations
